# Pilot study of using transcranial temporal interfering theta-burst stimulation for modulating motor excitability in rat

**DOI:** 10.1186/s12984-024-01451-5

**Published:** 2024-08-30

**Authors:** Chun-Wei Wu, Bor-Shing Lin, Zhao Zhang, Tsung-Hsun Hsieh, Jian-Chiun Liou, Wei-Lun Lo, Yu-Ting Li, Shao-Chu Chiu, Chih-Wei Peng

**Affiliations:** 1 School of Biomedical Engineering, College of Biomedical Engineering, Taipei Medical University, Taipei, 11031 Taiwan; 2 Department of Computer Science and Information Engineering, National Taipei University, New Taipei City 237303, Taiwan; 3 School of Mechanical and Electrical Engineering, Wuyi University, Wuyishan City, Fujian Province China; 4 School of Physical Therapy and Graduate Institute of Rehabilitation Science, College of Medicine, Chang Gung University, Taoyuan, Taiwan; 5 Neuroscience Research Center, Chang Gung Memorial Hospital, Linkou, Taiwan; 6 Department of Surgery, Division of Neurosurgery, Shuang Ho Hospital, Taipei Medical University, New Taipei City, Taiwan; 7 Department of Surgery, School of Medicine, College of Medicine, Taipei Medical University, Taipei, Taiwan; 8 Taiwan Instrument Research Institute, National Applied Research Laboratories, Hsinchu, Taiwan; 9 School of Gerontology and Long-Term Care, College of Nursing, Taipei Medical University, Taipei, Taiwan; 10 Graduate Institute of Biomedical Optomechatronics, College of Biomedical Engineering, Taipei Medical University, Taipei, 11031 Taiwan

**Keywords:** Brain stimulation, Transcranial temporal interference stimulation, Theta burst stimulation, Transcranial electrical stimulation, Neuroplasticity, Motor evoked potential

## Abstract

Transcranial temporal interference stimulation (tTIS) is a promising brain stimulation method that can target deep brain regions by delivering an interfering current from surface electrodes. Most instances of tTIS stimulate the brain with a single-frequency sinusoidal waveform generated by wave interference. Theta burst stimulation is an effective stimulation scheme that can modulate neuroplasticity by generating long-term potentiation- or depression-like effects. To broaden tTIS application, we developed a theta burst protocol using tTIS technique to modulate neuroplasticity in rats. Two cannula electrodes were unilaterally implanted into the intact skull over the primary motor cortex. Electrical field of temporal interference envelopes generated by tTIS through cannula electrodes were recorded from primary motor cortex. Theta burst schemes were characterized, and motor activation induced by the stimulation was also evaluated simultaneously by observing electromyographic signals from the corresponding brachioradialis muscle. After validating the stimulation scheme, we further tested the modulatory effects of theta burst stimulation delivered by tTIS and by conventional transcranial electrical stimulation on primary motor cortex excitability. Changes in the amplitude of motor evoked potentials, elicited when the primary motor cortex was activated by electrical pulses, were measured before and after theta burst stimulation by both techniques. Significant potentiation and suppression were found at 15 to 30 min after the intermittent and continuous theta burst stimulation delivered using tTIS, respectively. However, comparing to theta burst stimulations delivered using conventional form of transcranial electrical stimulation, using tTIS expressed no significant difference in modulating motor evoked potential amplitudes. Sham treatment from both methods had no effect on changing the motor evoked potential amplitude. The present study demonstrated the feasibility of using tTIS to achieve a theta burst stimulation scheme for motor cortical neuromodulation. These findings also indicated the future potential of using tTIS to carry out theta burst stimulation protocols in deep-brain networks for modulating neuroplasticity.

**Supplementary Information:**

The online version contains supplementary material available at 10.1186/s12984-024-01451-5.

## Introduction

Brain stimulation can modulate brain activities by altering the neural membrane potential or action potential. Additionally, it can lead to long-lasting after-effects such as long-term potentiation or depression (LTP/LTD). This characteristic makes brain stimulation a useful tool for investigating brain-behavior relationships and a potential therapeutic approach for neurodegenerative diseases [1].

One specific brain stimulation protocol is theta burst stimulation (TBS), which involves repetitive transcranial magnetic stimulation (rTMS) and can induce various effects on motor excitability depending on the type of TBS applied [2–4]. Notably, Huang et al. were pioneers in applying TBS protocols to the human primary motor cortex (M1). They observed that intermittent (i)TBS enhanced motor excitability, while continuous (c)TBS suppressed it in a sustained manner [2]. This bidirectional modulation of motor excitability highlights the promising potential of TBS as a valuable tool for studying brain neuroplasticity and as a therapeutic intervention for addressing motor deficits in neurological disorders [3, 4].

The pattern of TBS plays a crucial role in determining the direction of change in motor excitability. TBS is characterized by short pulses (with pulse widths of < 1 ms) arranged in bursts, with bursts repeating at a theta frequency of 5 Hz. Each burst consists of three pulses delivered at a rate of 50 Hz. Specific TBS protocols, namely cTBS and iTBS, produce distinct effects on motor plasticity [2]. For the cTBS protocol, which induces LTD-like effects, 5-Hz bursts are continuously delivered. In contrast, the iTBS protocol, producing LTP-like effects, involves delivering 5-Hz bursts for 2 s alternating with an 8-s rest [2].

To evaluate the modulatory effects on motor plasticity after a TBS intervention, researchers commonly assess changes in motor-evoked potential (MEP) amplitudes [5]. To activate the corticospinal tract in the M1, a single-pulse stimulation is applied. This stimulation leads to excitation of the downstream target muscle, generating an MEP with a certain latency after stimulation. The MEPs are recorded in the target muscle using electromyography (EMG), and their amplitudes, which reflect the excitability of the M1, are measured as peak-to-peak amplitudes [5].

Neuromodulation of cortical excitability by TBS has been widely studied [2–4, 6–11]. The potential mechanism of TBS exerting bidirectional neuroplastic modulation in post-synaptic neurons was discussed in recent review articles [4, 6, 9–11]. iTBS and cTBS are believed to be able to trigger different patterns of post-synaptic Ca^2+^ dynamics through glutamine and N-methyl-d-aspartate (NMDA) receptor pathways that lead to LTP or LTD [4, 6]. And later, the role of hierarchy of pyramidal projections in different layers of the motor cortex (layers 2/3 and 5) and the role of GABAergic interneurons were taken into consideration [9–11]. In a brief summary, interactions of glutamatergic and GABAergic neurotransmissions in cortical networks cooperate with post-synaptic Ca^2+^ dynamics that eventually determine the direction of neuroplastic modulation [9–11].

In addition to using rTMS, TBS has been applied to modulate M1 excitability using various methodologies. Previous animal studies demonstrated the modulatory effects of M1-TBS on MEP amplitudes using cortical electrical stimulation (CES) [7, 8, 12, 13] and optogenetic stimulation [8]. Moreover, M1-TBS using CES enhanced functional recovery from animal models of stroke [14], traumatic brain injury [15], and Parkinson’s disease (PD) [16]. Besides M1 modulation, TBS using deep-brain stimulation (DBS) targeting various subcortical areas also showed therapeutic potential. TBS delivered to the globus pallidus internus increased the theta frequency power in the dorsolateral prefrontal cortex with good tolerance in PD patients [17]. Later, TBS applied to the subthalamus nucleus was demonstrated to be effective in clinical PD symptom reduction without serious adverse events [18, 19]. Meanwhile, TBS delivered to the motor thalamus using deep-brain optogenetic stimulation improved akinesia in parkinsonian rats [20, 21]. TBS applied to the fornix, the efferent tract of the hippocampus, showed the ability to enhance visual-spatial memory in a clinical study with four participants implanted with DBS [22]. In summary, applying TBS with DBS that can target various deep-brain regions revealed clinical significance. However, invasive DBS requires a craniotomy that generates risks and limits its use. Using a non-invasive way to deliver a TBS scheme for deep-brain neuromodulation seems to be a promising and logical approach.

Transcranial temporal interference (TI) stimulation (tTIS) is a novel brain stimulation technique using an interfering electric field to stimulate superficial or deep-brain neurons [23, 24]. This approach involves application of two sets of high-frequency sinusoidal currents (≥ 1 kHz, which alone are insufficient to activate neurons) with a difference in low-frequency (Δf: usually 1–50 Hz) outside the brain. As a result, a TI envelope modulated at Δf is generated in the target area within the brain [23]. By appropriately configuring the electrode montage, the TI envelope can selectively stimulate deep-brain regions while leaving superficial layers unaffected.

Many computational models have been performed to investigate TI envelope generation inside the brain for determining the electrode montage and current parameters [25–36]. And the efficacy of tTIS in regulating neuron firing was reported in recent animal studies. By detecting c-*fos* expression, tTIS was demonstrated to activate hippocampal neurons of the mouse without recruiting the overlying cortex [23]. Another study used tTIS to target CA3 of the mouse hippocampus to focally evoke seizure-like events [37]. Later, tTIS from the hippocampus was reported to suppress epileptic markers in mice [38] and swine [39]. In the case of tTIS-induced M1 activation, some reported that M1-tTIS is capable of inducing a myoelectrical response in EMG signals [40, 41], and inducing movements of the forelimbs [41, 42]. Furthermore, activation of the left or right forelimb muscles is steerable through adjusting the ratio of current intensities without physically relocating the electrodes [23]. In summary, those animal studies demonstrated the feasibility of tTIS to locally regulate neural firing in the brain with spatial specificity.

A few studies explored the safety and efficacy of tTIS in the healthy human brain. Those studies usually employed a current intensity of 2 mA in a single channel (peak-to-peak 4 mA for two channels totally), stimulation frequencies of 6/20/70 Hz, and a stimulation duration of 20 ∼ 30 min [43–45]. Side effects occurring during tTIS stimulation were minor and tolerable, and no serious or intolerable adverse effects were reported when tTIS was implemented in healthy younger adults [43–45]. As for the treatment efficacy, a study compared the effect of 20-Hz M1-tTIS and M1-transcranial direct current stimulation (tDCS) on functional connectivity in 40 healthy participants. Results showed that both 20-Hz M1-tTIS and M1-tDCS significantly enhanced resting-state functional connectivity between M1 and the secondary motor cortex (premotor cortex and supplementary motor cortex), and the enhancement may have been related to motor functions [46]. Another study involving 24 healthy participants demonstrated that 70-Hz M1-tTIS could reduce the reaction time and enhance the excitability of M1. Meanwhile, 20-Hz M1-tTIS facilitated motor learning, which was significantly positively correlated with an increase in the MEP [44]. Furthermore, in a randomized controlled, single-blinded pilot study, 60 participants were randomly assigned to receive 6-Hz tTIS and transcranial alternating current stimulation (tACS) targeting the right frontoparietal cortex. Results demonstrated that working memory under high-load cognitive tasks appeared to be slightly improved by tTIS compared to tACS-sham [45]. In summary, limited evidence suggests that tTIS may be an effective and safe method for modulating motor excitability, motor network activity, and working memory in healthy participants. However, for patients with neurological diseases or disorders, further research is needed to explore the potential efficacy and safety of tTIS in clinical applications.

To date, tTIS has predominantly been applied at constant stimulation frequencies. However, its suitability for complex paradigms like TBS remains unclear. In this study, we explored the feasibility of utilizing tTIS to deliver TBS protocols for M1 neuromodulation. First, we generated in vivo TBS schemes using the tTIS device developed in our previous work [41]. Two independent currents with kilohertz frequency were both delivered into single pair electrode on the skull to generate interfering waveform. We utilize this pre-modulation approach to produce the waveform of TBS in the M1 region. Subsequently, we evaluated the neuromodulatory effects of TBS delivered via tTIS by measuring changes in the amplitude of MEPs generated when the M1 was stimulated. The findings from this investigation have the potential to open new avenues for non-invasive tTIS-TBS applications in deep-brain neuromodulation.

## Materials and methods

### Animals

Animal experiments were reviewed and approved by the Institutional Animal Care and Use Committee of Taipei Medical University (IACUC approval no. LAC-2019-0518). Seventeen male Sprague-Dawley rats (BioLASCO Taiwan, Taipei, Taiwan) weighing 300 ∼ 350 g were utilized in this study. All animals were maintained in an animal house with constant temperature (22 ± 2 °C) and humidity (55 ± 10%). A 12-h light/dark cycle with food and water available ad libitum was applied to all animals. At the end of the study, animals were sacrificed using carbon dioxide ventilation followed by cervical dislocation.

### Implantation of electrodes

**Fig. 1:**
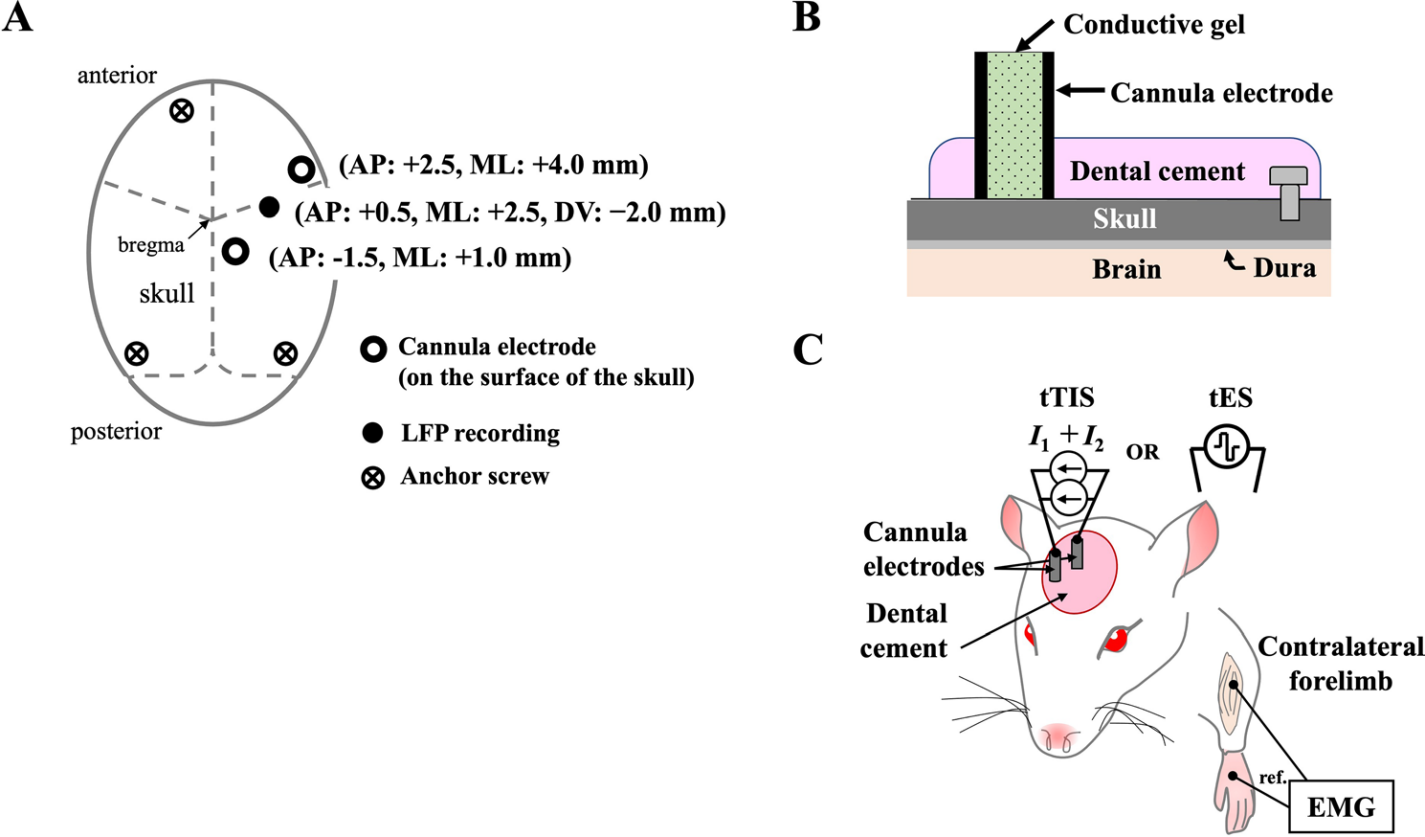
Experimental set-up of the study. (**A**) The diagram shows the arrangement of the cannula electrodes, burr hole for electric field potential recording and anchor screws on the skull. (**B**) The diagram shows the cannula electrode fixed to the surface of the skull using dental cement and anchor screws. (**C**) The primary motor cortex (M1) was stimulated by delivering the transcranial temporal interference stimulation (tTIS) or transcranial electrical stimulation (tES) through the 2-pole cannula electrode pair. Activation of M1 was evaluated using EMG from the brachioradialis in the contralateral forelimb

The cannula electrodes used to stimulate the M1 were implanted following a previous protocol [41]. Rats were anesthetized with inhalation of 4% isoflurane in O_2_, the head was then placed in a stereotaxic apparatus (Model 902, David Kopf Instruments, Tujunga City, CA, USA), and anesthesia was maintained with 2.5% isoflurane inhalation. An incision was made on the scalp to expose the skull after the soft tissue had been removed with a 3% hydrogen peroxide solution. Two cannula electrodes made of stainless-steel tubes cut from 16G needles (O.D. 1.7 mm; I.D. 1.2 mm; length 10.0 mm) were placed on the surface of the intact skull above the right M1. The positions relative to the bregma were anteroposterior (AP) 2.5 mm, mediolateral (ML) 4.0 mm, and AP − 1.5 mm and ML 1.0 mm (Fig. 1A). A burr hole was drilled through the skull (AP 0.5 mm; ML 2.5 mm) to record electrical field potentials induced by tTIS. Three additional burr holes were drilled around the surgical opening to accommodate the placement of anchor screws (length 3.0 mm; width 1.4 mm). The cannula electrodes were fixed to the skull by covering them with dental cement (Fig. 1B). The experiments were performed 1 week later to allow wound healing. Before the experiment, the cannula electrodes were filled with conductive gel (SignaGel, Parker Laboratories, Fairfield, NJ, USA). The tTIS or transcranial electrical stimulation (tES) was delivered through the cannula electrodes to modulate motor excitability, which was further evaluated via contralateral forelimb MEPs (Fig. 1C).

### Generation of TBS scheme

TBS protocols in this study were applied using two types of transcranial stimulation: tES and tTIS. First, TBS was delivered using tES [7, 8]. A burst was composed of three continuous biphasic pulses with a 1-ms pulse width and 20-ms pulse interval (50 Hz) using a pulse stimulator (model 2100, A-M Systems, Sequim, WA, USA). For the continuous (c)TBS protocol (tES-cTBS), bursts were continuously delivered at 5 Hz (cycle: 200 ms) for 40 s (for a total of 200 bursts/600 pulses). For the intermittent (i)TBS protocol (tES-iTBS), 5-Hz bursts were delivered for 2 s followed by a rest of 8 s for 20 cycles (for a total of 200 bursts/600 pulses). For the sham control (tES-sham), no electrical pulse was delivered. Second, pre-modulated TBS schemes were generated and delivered through 2-pole montage to target the surface of the cortex using a previously developed dual-channel high-frequency electrostimulator [41]. The device can output two discontinuous sinusoidal currents in a specific temporal manner to generate discontinuous TI envelopes that mimic TBS. For the cTBS protocol (tTI-cTBS), 5-Hz bursts were continuously delivered for 40 s (for a total of 200 bursts/600 envelopes). For the iTBS protocol (tTI-iTBS), 5-Hz bursts were delivered for 2 s followed by a rest of 8 s for 20 cycles (for a total of 200 bursts/600 envelopes). As for the sham control (tTI-sham), two currents at the same modulating frequency (2000 Hz) that generated no TI envelope were applied.

### Recording of the electric field potential generated by tTIS

Generation of tTI-TBS scheme inside the brain was verified by electrical field potential recordings. Rats were anesthetized as previously described. When the skull was exposed and a burr hole was drilled, a concentric recording electrode (SS80SNE-10; Microprobes for Life Science, Gaithersburg, MD, USA) was inserted through the burr hole beneath the surface of the cortex (AP 0.5 mm; ML 2.5 mm; DV 2.0 mm). The current envelopes were recorded during two-pole tTI-TBS with a sampling rate of 25 kHz (MP36, BIOPAC System, Goleta City, CA, USA). The signal was amplified 100-fold followed by a 60-Hz notch filter and a 1.5 ∼ 12 kHz bandpass filter. Amplitudes of the crests and troughs of the tTIS current envelopes (mV) were plotted.

### Motor evoked potentials (MEPs)

Activation of the motor cortex was observed using MEPs following previous methods [7, 8]. Rats were intraperitoneally anesthetized with 50 mg/kg of a dissociative anesthetic (Zoletil, Vibac, Carros, France) and 10 mg/kg xylazine (Bayer, Leverkusen, Germany) 30 min prior to the experiment. MEPs were elicited by repeated biphasic electrical pulses through the cannula electrode pair (pulse width 1 ms; pulse interval 10 s: pulse intensity 0.1 ∼ 10.0 mA) using a pulse stimulator (model 2100, A-M Systems, Sequim, WA, USA). EMG signals were simultaneously collected using 27G stainless-steel needle electrodes inserted into the brachioradialis muscles in the contralateral forelimb. Reference electrodes were inserted into the paws (Fig. 1C). The ground electrode was inserted into the base of the tail. The signal was amplified 2000-fold before a 60-Hz power-line notch filter, then a bandpass filter with a 0.5 ∼ 500-Hz cutoff frequency was further adopted to eliminate motion artifacts and stimulation artifacts generated by the carrier frequencies of tTIS. The EMG trace was sampled at 10 kHz and plotted (MP36, BIOPAC System). The MEP amplitude was determined by measuring the peak-to-peak amplitude. The minimal intensity of the stimulation required to induce an MEP of greater than 20 mV was defined as the resting motor threshold (RMT). The RMT for tTIS and tES were determined separately.

### Experimental design

The modulatory effects of tTI-TBS or tES on motor excitability were evaluated by changes in MEPs. MEPs were induced by 0.1 Hz tES at an intensity of 120% of the RMT. Average amplitudes of MEPs were calculated from every 5 min of recording (30 measurements in 5 min). Thirty minutes after anesthesia, MEPs were measured for 10 min as a baseline. Then six types of interventions were applied over M1 through the cannula electrode pair at an intensity of 80% of the RMT (Fig. 3A). Both types of TBS (iTBS and cTBS) and sham treatments were applied using tTIS and tES. After the intervention, MEPs were recorded for another 30 min. Fold changes of MEP amplitudes were calculated as the ratio over the average baseline at 5 min before treatment. Five days of resting between each experiment eliminated any effects of the anesthetic and TBS intervention in the rats.

### Data analysis

Data were analyzed and are presented using GraphPad Prism (vers. 5.01, GraphPad Software, Boston, MA, USA) with statistical significance set to *p* < 0.05. The normality of the samples was first tested using Shapiro-Wilk and Kolmogorov-Smirnov test (a = 0.05). If any of the sample did not pass the normality test, non-parametric methods were adopted for the presentation and statistical analysis of the results. Quantitative data are presented using boxplots (minimum, first quartile, medium, third quartile, and maximum) while “+” indicates the arithmetic mean. MEPs were normalized to the baseline recorded at 5 min before treatment (–5 min). Non-parametric multiple comparisons were performed to separately analyze the effects of two factors on MEP activities. First, a Kruskal-Wallis test was used to verify any significant difference among the iTBS, cTBS, and sham treatment at each time course. Second, Friedman’s test was used to examine the statistical significance from repeated measurements of each time course. Significant differences between each time course versus the baseline (–5 min) were further identified by a post-hoc Dunn’s test: * *p* < 0.05. To further analyze the difference between tES-iTBS versus tTI-iTBS, and tES-cTBS versus tTI-cTBS at each time point, the Mann-Whitney test was used with significant level of a *=* 0.05.

## Results

### Generation of the TBS scheme using 2-pole tTIS and its effects on M1 activation

**Fig. 2:**
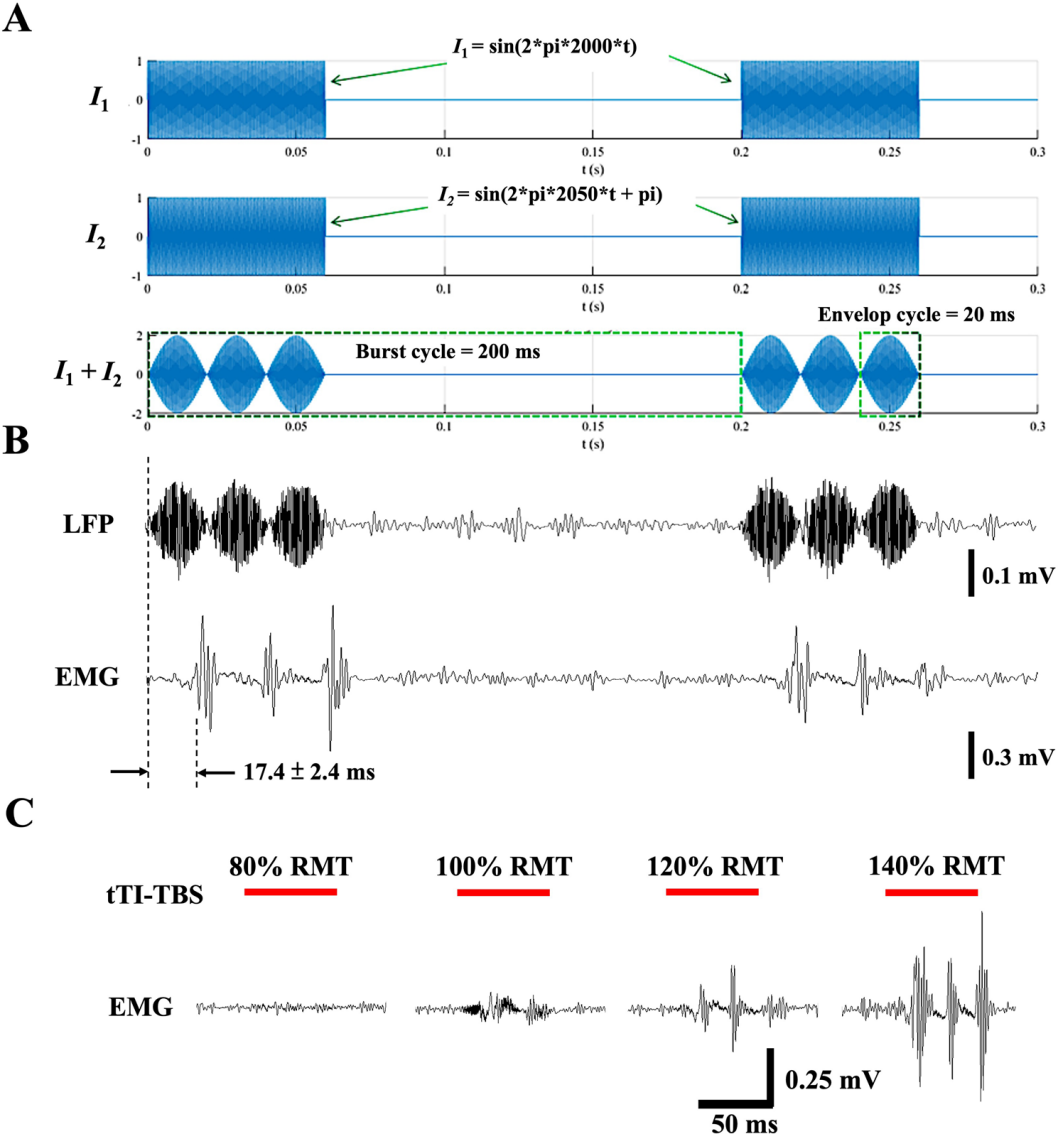
Primary motor cortex (M1) activation by transcranial temporal interference (tTI)-theta burst stimulation (TBS). **A.** Synthesis of tTI-TBS from two channels of currents with specific temporal modulations. **B**. M1 activation by tTI-TBS was observed using the recording of electric field potential and EMG. **A** and **B** have the same time scale. **C.** Dose-dependent responses in motor evoked potentials (MEPs) induced by tTI-TBS delivered to M1 with various intensities (S2). Red bars indicate the duration of TBS

Generation of the TBS scheme in M1 using tTIS technology was verified by recording of the electric field potential in cortical tissue. The synthetic tTI-TBS envelopes were demonstrated by computing the summation of two sinusoidal signals (*I*_1_: 2000 Hz and *I*_2_: 2050 Hz) with a 60/120-ms on/off cycle. Three envelopes repeated at 50 Hz were generated during the ‘on’ time, and the burst composed of three envelops was repeatedly generated at 5 Hz (with a 200-ms interval) (Fig. 2A). When *I*_1_ and *I*_2_ were delivered to the cortical surface through transcranial electrodes in the 2-pole mode, the pre-modulated TBS scheme (S1) was observed in electric field potential traces from M1 which were identical to a synthetic TBS scheme (Fig. 2B, upper trace). The TBS scheme generated in M1 further led to activation of the corresponding forelimb muscle. An EMG signal extracted from the contralateral brachioradialis muscle showed synchronized MEPs with a latency of 17.4 ± 2.4 ms (Fig. 2B, lower trace). An intensity-dependent increase was observed in amplitudes of MEPs when the intensity of tTI-TBS increased from 80 to 140% of the RMT (Fig. 2C & S2). When using tTI-TBS to induce MEPs, the average RMT was determined as 3.5 ± 1.2 mA (peak-to-peak values of the sinusoidal wave; *n* = 7) for both *I*_1_ and *I*_2_. The injection current intensity for *I*_1_ and *I*_2_ to generate cortical field potential and simultaneous MEPs (Fig. 2B) are both 4.2 mA (peak-to-peak values of the sinusoidal wave), which equals to 120% RMT. As for the later tTI-TBS intervention using 80% RMT intensity, which equals to 2.8 ± 0.9 mA for both *I*_1_ and *I*_2_. The total injection current intensity will be the summation of *I*_1_ and *I*_2_.

### After-effects of TBS interventions using tTIS and tES methods on M1 excitability

**Fig. 3:**
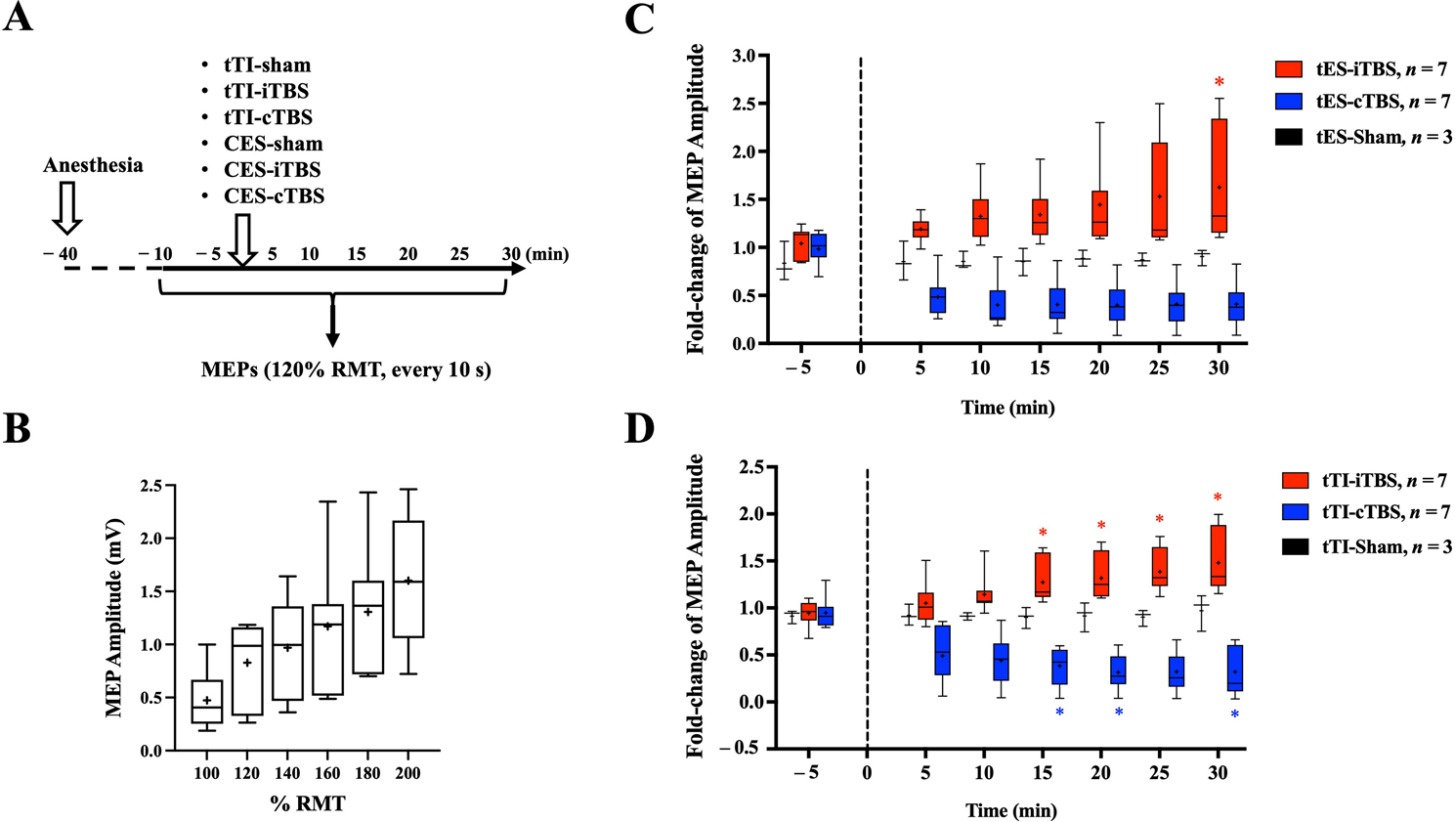
Neuromodulation of primary motor cortex (M1) excitability. **A**. The diagram demonstrates the experimental design of the study. The after-effects of transcranial temporal interference stimulation (tTIS) and transcranial electrical stimulation (tES) on M1 excitability were determined by evaluating the change in the motor evoked potential (MEP) amplitude after the intervention versus the baseline. **B**. MEP responses under various intensities of tES of M1. Boxplot summarizes the MEP peak-to-peak amplitudes induced by a tES single pulse at 100%∼200% resting motor threshold (RMT) from seven rats. **C**. Relative changes in the MEP amplitude induced by M1-tES in the form of intermittent theta burst stimulation (iTBS), continuous (c)TBS, and sham treatment. Boxplot shows fold-changes of MEP amplitudes at various time courses. **D**. Relative changes in the MEP amplitude induced by M1-tTIS in the form of iTBS, cTBS, and sham treatment. Boxplot shows fold-changes of MEP amplitudes at various time courses. The vertical dotted line indicates the time-point of intervention. **+**: mean; *: *p* < 0.05 versus the baseline at 5 min before treatment began

An MEP input-output curve was used to test the sensitivity of cortical modulation by transcranial stimulation via the cannula electrode interface. A dose-dependent increase was observed in MEP amplitudes when the tES pulse stimulation intensity increased from 100 to 200% of the RMT (Fig. 3B). A significant correlation between the stimulation intensity and MEP amplitudes was confirmed by computing nonparametric Spearman correlations (Spearman’s rank correlation coefficient = 1, *p* < 0.01). When using tES pulse to induce MEPs, the average RMT was determined as 2.9 ± 1.0 mA (peak-to-peak values of the biphasic square pulse; *n* = 7). Therefore, for the later tES-TBS intervention using 80% RMT intensity, which equals to 2.2 ± 0.8 mA.

Modulation of M1 neuroplasticity was first tested using tES-TBS methods to compare with previous finding [7, 8]. We have tested the normality of the samples using Shapiro-Wilk test and Kolmogorov-Smirnov test, and some groups were against null hypothesis that data are normally distributed when a = 0.05 (S3). Therefore, we were using non-parametric methods for statistical analysis. MEP changes before and after tES-iTBS, tES-cTBS, and tES-sham treatments were evaluated (Fig. 3C). Results showed a trend of potentiation in MEP amplitudes after tES-iTBS treatment, and it reached statistical significance at 30 min after treatment (Friedman’s test followed by post-hoc Dunn’s test, *p* < 0.05). A trend of depression was also found in MEP amplitudes after tES-cTBS treatment; however, no statistical significance was found. For the effect of the tTI-TBS protocol on motor plasticity, our results showed significant potentiation in MEP amplitudes 15 min after tTI-iTBS treatment, and a significant depression in MEP amplitudes 15 min after tTI-cTBS treatment versus the baseline (Friedman’s test followed by post-hoc Dunn’s test, *p* < 0.05). In both the tES and tTIS experiments, no noticeable change was observed after sham treatments. To further compare the different effect between tES-iTBS versus tTI-iTBS, and tES-cTBS versus tTI-cTBS on MEP amplitudes at each time point, the Mann-Whitney test was performed with significant level set to 0.05 (S4). Supplementary results show that there is no significant difference between the effects of using tTIS and tES interventions on MEP modulation at any of the time point.

## Discussion

In this pilot animal study, the feasibility of using tTIS to carry out a TBS protocol was tested by modulating M1 neuroplasticity. First, the TBS scheme generated by two-pole tTIS was confirmed by direct measurement of the field potential inside M1. Each burst consisted of three tTIS envelopes that were similar to the traditional TBS scheme. Each envelope elicited a corresponding MEP response as monitored by EMG from the contralateral brachioradialis muscle. These MEPs were specifically generated by M1 activation since an intensity-dependent response was observed when the intensity of tTI-TBS changed. Then, effects of tTI-TBS on M1 excitability were tested by measuring relative changes in MEP amplitudes before and after the interventions. Both tES-TBS and tTI-TBS can modulate MEP activities. When compared to tES-TBS, tTI-TBS showed no significant difference in modulation of M1 neuroplasticity. Variation in MEPs after tES-TBS intervention seems higher than those after tTI-TBS intervention. We cannot conclude the cause of higher variability in MEP amplitudes after tES-TBS intervention from current data. We speculate that the lot-to-lot variation of the anesthetic drugs and differences in stimulation equipments (tES versus tTIS) may contribute to this observation. In sum, these studys demonstrated the potential of using tTIS to carry out a complex stimulation paradigm such as a TBS scheme, instead of a single-frequency sinusoidal waveform that was used in previous tTIS studies. To our knowledge, this is the first study to demonstrate the feasibility of using the tTIS technique to carry out a TBS scheme in an animal model. The long-term effects of brain neuromodulation when using tTI-TBS on the motor excitability nevertheless warrant further exploration.

A major limitation of the present study is that tTIS was delivered in a semi-invasive manner through electrodes directly attached into the intact skull, instead of the scalp to achieve complete non-invasiveness. Previous studies demonstrated significant electric field loss due to the shunting effects of the skin and soft tissues [47–49]. Over an approximately 4-fold decrease in the intracerebral electric field was observed when the electric current was delivered through the scalp compared to the skull. Therefore, to better demonstrate the concept of using tTIS to achieve a TBS scheme, we used skull electrodes instead of scalp electrodes to stimulate M1. For totally non-invasive applications in the future, further study is needed to investigate the shunting effects of the skin and soft tissues on generating a TBS scheme by tTIS via scalp electrodes.

Another significant limitation of the study was the arrangement of the electrodes. A 4-pole montage, that separately delivers the two currents through two set of electrode pairs (four electrodes), was used in several tTIS animal studies because it can target deep-brain areas without recruiting the superficial cortex above it [23, 37, 38, 40, 42]. A previous study showed a 2-pole montage, that delivered two currents through one electrode pair, generated a significant interfering envelope potential to the superficial cortex while the 4-pole montage targeted cortical and subcortical areas [41]. In the current pilot study, the aim was to specifically target M1 to demonstrate the modulatory effect of tTI-TBS on M1 neuroplasticity. Therefore, a 2-pole montage was adopted in this study for better recruitment of M1 compared to using a 4-pole mode. However, the 2-pole stimulation is a form of pre-modulated stimulation that directly delivers the interfering waveform to the tissue between two electrodes, primarily targeting the surface of the cortex. When considering the application of the 2-pole montage in humans, it is crucial to recognize that the impact of the skull-to-brain distance may result in varying effects compared to rats, particularly concerning the modulation percentage in the target area and the stimulation of unintended regions.

When targeting motor cortex, a study demonstrated that the thalamus contributes to the late evoked potential (EP) component elicited by motor cortical stimulation [50]. They have further demonstrated that the magnitude of this late EP component correlates with the activity of thalamic neurons, modulated by the subject’s behavioral state [50]. This finding indicates that cortico-thalamic-circuit engaged by cortical stimulation is modulatable. In our current study, the tTI-TBS generates burst current in M1 and the stimulation artifact is too strong to observe the cortical EP. The question of whether the activity of the thalamus is also modulated by tTI-TBS when it alters the excitability of the motor cortex remains undiscovered. Further study using 4-pole tTI-TBS to target thalamus is required to unravel the role of cortico-thalamic-circuit in. neuroplasticity.

Another feature of using pre-modulated tTIS and 2-pole montage is the lack of onset effect. When two high-frequency currents are delivered independently (4-pole montage), the sudden onset of the stimulation may generate neural response. Previous studies have employed ramping up and down of stimulation current amplitude at its onset and offset to prevent this effect [23]. In our current 2-pole montage set-up, two high-frequency currents are pre-modulated into a spindle-shaped TBS waveform (S1) before they are delivered. We speculate that the pre-modulation can prevent the sudden initiation of the stimulation. Thus, no response was observed at the moment of currents onset in electric field potential trace and EMG recording (Fig. 2B).

The TI stimulation might require a higher current intensity to achieve equivalent effects as CES [51]. In the study, we have reported RMT of 3.5 ± 1.2 mA (peak-to-peak values of the sinusoidal wave) for both currents of tTIS. After summation, the total current intensity is around 7.0 mA. This RMT for tTIS is much higher than RMT of 2.9 ± 1.0 mA for tES pulse (peak-to-peak values of the biphasic square pulse).

An important challenge for tTIS is the assumption that neurons do not response to individual unmodulated high frequency waveforms in non-target areas [6]. However, research has shown that neurons do respond to high-frequency electric fields and lead to inhibition of action potential propagation, also known as conduction block [52]. This phenomenon might have significant implications for considering the neuromodulation effect of tTIS. In our current study, the tTIS was delivered in the form of pre-modulation through 2-pole montage (Fig. 2A&B, S2). This set-up has prevented the delivering of unmodulated high frequency component in non-target areas. When using 4-pole or multiple-channel montages to employ tTIS for targeting subcortical tissues, computational study and field potential measurement in deep-brain tissues may help to explore the feasibility and the potential mechanisms behind it.

In sum, the current study does not provide a method for implementing TBS protocols using a typical 4-pole temporal interference montage. Potential issues with such a setup include onset effects and conduction block. Addressing these issues would require further research. Thus, the study only involves using amplitude-modulated TBS stimulation with a 2-pole setup to mimic the electric field patterns generated by typical 4-pole temporal interference in brain tissue and to explore its effects on neural plasticity.

## Conclusions

This pilot study demonstrated the feasibility of using tTIS to achieve a TBS scheme for M1 neuromodulation. The modulatory effects of tTI-TBS on neuroplasticity were more significant than the effects derived from conventional tES-TBS protocols. These results show the potential of using tTIS to generate a complex stimulation paradigm such as TBS schemes, for neuromodulation in certain brain networks. Further study is required to verify the efficacy of tTI-TBS with multiple channels and non-invasive scalp electrodes on deep-brain neuromodulation for future translational medicine.

### Electronic supplementary material

Below is the link to the electronic supplementary material.

Supplementary Material 1

## Data Availability

Available upon request.

## Abbreviations

AP: Anteroposterior
CES: Cortical electrical stimulation
cTBS: Continuous TBS
DBS: Deep-brain stimulation
EMG: Electromyography
iTBS: Intermittent TBS
LTP/LTD: Long-term potentiation/depression
M1: Primary motor cortex
MEP: Motor evoked potential
ML: Mediolateral
NMDA: N-methyl-d-aspartate
PD: Parkinson’s disease
RMT: Resting motor threshold
rTMS: Repetitive transcranial magnetic stimulation
tACS: Transcranial alternating current stimulation
TBS: Theta burst stimulation
tDCS: Transcranial direct current stimulation
tES: Transcranial electrical stimulation
tTI: Transcranial temporal interference
tTIS: Transcranial temporal interference stimulation
