# Supplementary material for: Pilot study of using transcranial temporal interfering theta-burst stimulation for modulating motor excitability in rat
Source: J Neuroeng Rehabil. 2024 Aug 30;21:147. doi: 10.1186/s12984-024-01451-5 (PMC11365202; doi:10.1186/s12984-024-01451-5)
Supplement: Supplementary file 1 — Supplementary Material 1 [file 12984_2024_1451_MOESM1_ESM.docx]

Supplementary table and figures


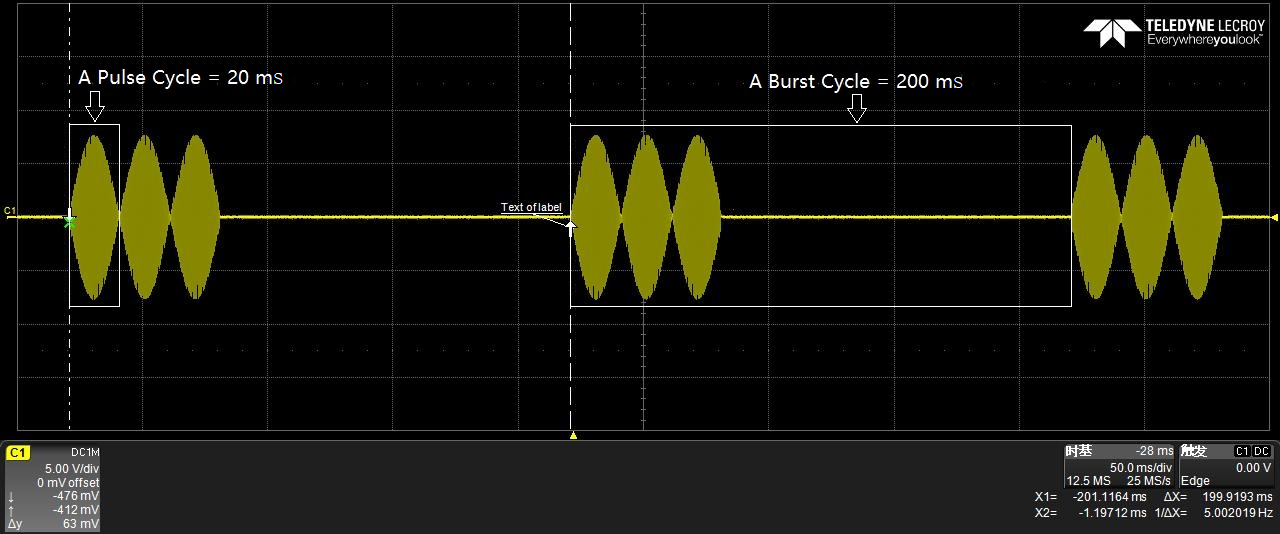


S1. Oscillograph of pre-modulated TBS waveform.

S2. MEP response generated by various intensities of tTI-TBS in M1. Boxplot summarizes the MEP peak-to-peak amplitudes induced by tTI-TBS at 100%~140% RMT (Figure 2C). Significant differences were confirmed using Kruskal-Wallis test followed by post-hoc Dunn's test. * *p* < 0.05; ** *p* < 0.001; + mean of the sample.

S3. Summary results of the sample groups that did not pass the normality test.

| Method | Sample group | *p* value | Passed normality test  (α = 0.05) |
| --- | --- | --- | --- |
| Shapiro-Wilk test | tTI-iTBS, 10 min | 0.0106 | No |
|  | tTI-iTBS, 15 min | 0.0171 | No |
| Kolmogorov-Smirnov test | tES-iTBS, 25 min | 0.0486 | No |
|  | tTI-iTBS, 10 min | 0.0234 | No |
|  | tTI-iTBS, 15 min | 0.0077 | No |


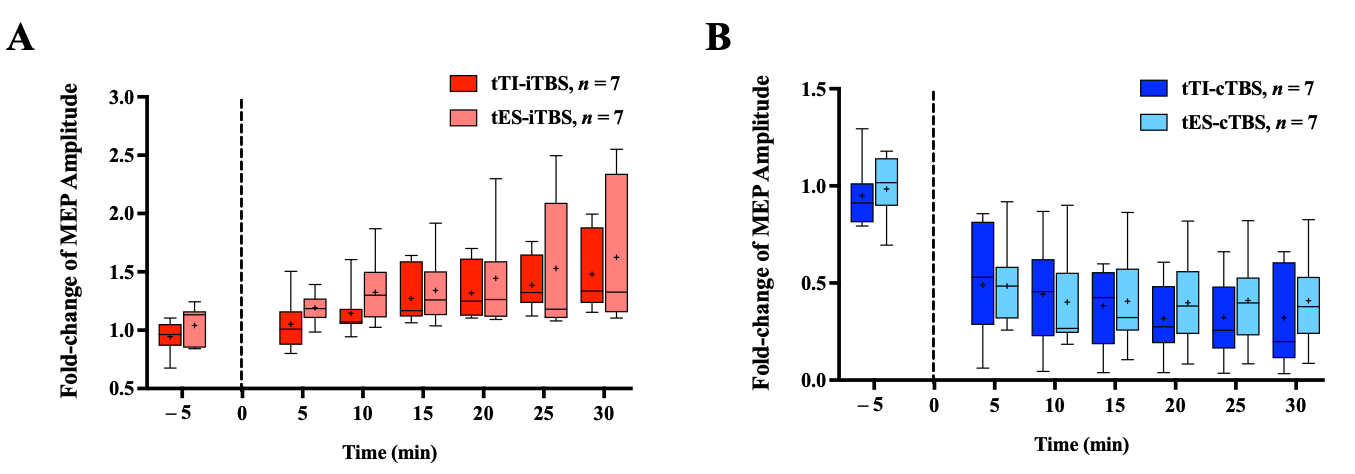


S4. Comparing the effect of TBS in the form of tTIS or tES on MEP amplitudes modulation. A. Relative changes in the MEP amplitude induced by tTI-iTBS versus tES-iTBS. B. Relative changes in the MEP amplitude induced by tTI-cTBS versus tES-cTBS. Boxplot shows fold-changes of MEP amplitudes at various time courses. The vertical dotted line indicates the time-point of TBS intervention. + mean of the sample; No statistical difference has been detected between tTI-TBS versus tES-TBS at any time point.
